# De Witt County Medical Society

**Published:** 1862-02

**Authors:** John Wright

**Affiliations:** Sec. pro tem.


### DE WITT COUNTY MEDICAL SOCIETY.

The Society met in Wapella on Tuesday, the 7th of January, 1862. The President called the Society to order at 10 o'clock A. M. The minutes of the previous meeting were read and approved. Society adjourned to meet at 2 P. M.

AFTERNOON SESSION.

Essays were called for. Dr. Shurtleff, the only essayist present, wished to be excused, on account of sickness.

Dr. Davis gave a verbal report of a case. Dr. Madden also gave a verbal report of a case. The cases reported elicited considerable discussion. Pneumonia, the subject for general discussion, was next called for. Dr. Wright opened the discussion by reading a paper on the diagnosis and treatment of Pneumonia. The discussion was continued until a late hour.

The following resolution was offered and adopted:

*Resolved*, That the thanks of the Society are due and are hereby tendered to Dr. Davis and lady, and Dr. Shurtleff for the excellent dinner gotten up for the Society.

Burns and scalds was the subject announced by the President for general discussion, at the next meeting of the Society. Drs. T. K. Edminston, W. W. Adams, B. K. Shurtleff and J. R. Richard, were continued as essayists. On motion, the proceedings of this meeting were ordered to be published in the *Central Transcript* and in the *Chicago Medical Journal*.

The Society then adjourned to meet in Clinton, the first Tuesday in April next. JOHN WRIGHT, *Sec. pro tem.*

